# The Effect of EBM Process Parameters on Porosity and Microstructure of Ti-5Al-5Mo-5V-1Cr-1Fe Alloy

**DOI:** 10.1155/2019/2903920

**Published:** 2019-04-01

**Authors:** Tomasz Kurzynowski, Marcin Madeja, Robert Dziedzic, Karol Kobiela

**Affiliations:** Faculty of Mechanical Engineering, Wroclaw University of Science and Technology, Wroclaw 50-370, Poland

## Abstract

In this article, the authors discuss the results of studies into the processing of Ti-5Al-5Mo-5V-1Cr-1Fe near-*β* titanium alloy (Ti-55511) by electron beam melting (EBM), an additive manufacturing technique. Due to its high flexibility in shaping mechanical properties, Ti-55511 alloy is commonly used in aircraft components such as landing gear or airframes. In this study, Ti-55511 powder was used and its properties were described as regards chemical composition and particle size distribution in order to assess its suitability for EBM processing and repeatability of results. 20 sets of processing parameters were tested in the energy input range between 10 J/mm^3^ and 50 J/mm^3^ (cathode current, 4.5 mA-19.5 mA; scanning speed, 1080 mm/s–23400 mm/s). Four types of top surfaces were obtained, namely, flat, orange peel, with single pores, and with swelling. Best results were obtained for the energy of 30 J/mm^3^: flat top surface and relative density in excess of 99.9%. Analysis of chemical composition showed that aluminum loss was below the specification minimum for the analyzed parameter sets. Scanning speed most significantly affected aluminum content: the lower the scanning speed, the higher the aluminum loss. Analysis of microstructures showed the dependence of lamellar *α*-phase volume fraction on the process parameters used. For low scanning speed, the determined *α*-phase volume accounted for about 78%. Higher scanning speed resulted in a decrease of the *α*-phase content to 61%. The dimensions of the lamellas and the amount of the *α*-phase strongly effected hardness results (360 HV to 430 HV).

## 1. Introduction

Titanium alloys are widely used in the aerospace, chemical, and medical industries due to their high relative strength and corrosion resistance. Ti-5Al-5Mo-5V-1Cr-1Fe alloy (Ti-55511) is a near-*β* alloy consisting of the *α*- and *β*-phases. It is used, among others, to manufacture parts exposed to high mechanical loads such as landing gear components, components of low-pressure compressor blades and fuel systems [1]. Ti-55511 alloy has high strength (1100-1260 MPa) and high fracture toughness (66-77 MPa·m^0.5^). It is suitable for long-term operation at temperatures up to 350-400°C and short-term operation at temperatures up to 750-800°C [2–4]. The mechanical properties of Ti-55511 are affected by morphology and amount of the *α*-phase. Therefore, tensile strength, ductility, and fatigue properties of the alloy can be modified by thermomechanical treatment [5–7]. This makes it possible to produce various types of the alloy microstructure, i.e., bimodal, equiaxed, or Widmanstätten type [8, 9].

In the conventional technique, a variety of thermomechanical operations are carried out to achieve the desired properties of parts made of Ti-55511 alloy. The parts are usually formed by forging which is expensive and time-consuming to implement as it requires tooling, dies, etc. to be prepared. It often takes months to create a die. In the case of discrete or low-volume production, tooling makes up for a significant percentage of the total cost of the part being produced. Therefore, electron beam melting (EBM), an additive manufacturing technique, shows great application potential in discrete or low-volume production as it reduces the lead time and cost of the part to be fabricated.

In EBM technology, a focused electron beam selectively melts a powder bed. Once the melting process on a single layer is completed, the build platform is lowered and a new layer of powder is deposited. The layer-by-layer process continues until the entire part is produced. Between melting and deposition of a new powder layer, electron beam heats powder surface to a temperature of about 750-850°C. This is to lightly sinter the powder and maintain elevated temperature of the build plate [10]. The elevated temperature reduces residual stresses in the produced parts. EBM processing provides finer microstructure of parts compared to casting or forging [11, 12]. EBM also allows to produce near-net shape parts, which may be an advantage in the processing of Ti-55511 alloy due to difficulty in its machining [1].

The aim of the studies outlined in this paper was to determine the influence of the main parameters of EBM process on the microstructure and properties of Ti-5Al-5Mo-5V-1Cr-1Fe alloy.

## 2. Materials and Methods

An Arcam A1 EBM machine is equipped with an electron beam gun of theoretical power of 3.5 kW. Electrons are emitted by a cathode made of tungsten wire and accelerated by 60 kV. The intensity of electron emission and hence the power required to melt the metal powder are controlled by the current supplied to the cathode. 15 × 15 × 15 mm specimens were produced, with a layer thickness of 50 *μ*m and a snake-like (zigzag) hatching strategy with a line offset of 100 *μ*m. After each layer, build platform was lowered by the nominal layer thickness, a new powder layer was applied, and hatching direction was rotated by 90°. Specimens were fabricated directly on a stainless steel substrate plate. The spot size of the electron beam was about 500 *μ*m in diameter in the focal point. The process was carried out at reduced pressure (2.1 × 10^−3^ mbar) which was controlled via the injection of helium (99.999% purity) to ensure proper working and stabilization of electron beam gun and also to avoid contamination. Powder surface temperature was maintained at 750-850°C.

**Figure 1:**
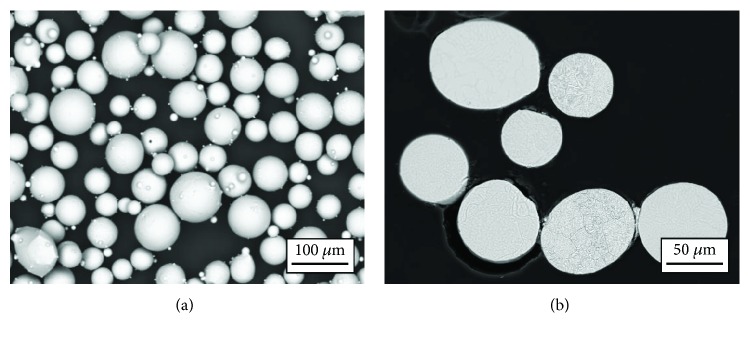
SEM images of Ti55511 powder: (a) general view and (b) cross sections; SEM-BSD detector.

Ti-55511 powder was prepared using atmospheric plasma spray (APS) technology. The powder particles were spherical with sparse satellites (small particles typical for APS technology) attached to them.  Figure 1(a) shows the SEM image of the surface of Ti-55511 powder particles used in the studies. Examinations of the particles' cross sections did not reveal internal pores, nonmetallic inclusions, or other material defects (Figure 1).

Table 1 compares the chemical composition of the powder specified by the manufacturer with the chemical composition specified by GOST 19807:1991.

**Figure 2:**
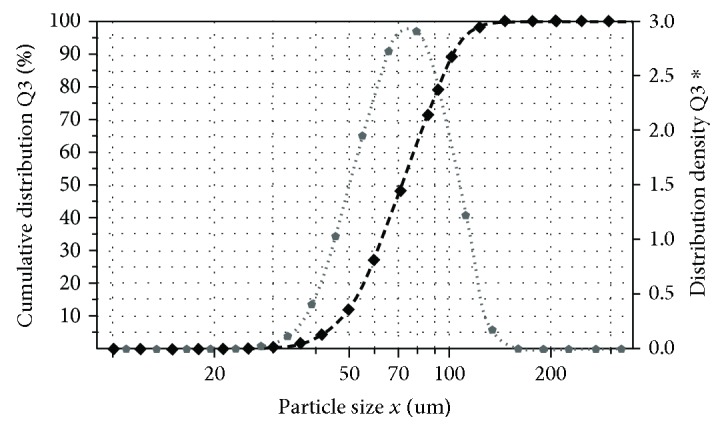
Analysis of size distribution of Ti-55511 powder fractions; dot line: cumulative distribution; dash line: distribution density.

The production of high-quality parts by EBM requires the use of a powder with a particle size between 45 and 106 *μ*m. The size of Ti-55511 powder particles was determined using the laser diffraction sizing technique with dynamic image analysis using the Q3 method (volume-base diameter). The results are shown in Figure 2.

As shown above, the average particle size of the powder for the 45-106 *μ*m range was *d*_50.Q3_ = 72.62 *μ*m. In EBM process, the theoretical thickness of the applied powder layer was 50 *μ*m. Heating consolidates the material as the grains are progressively linked. After the melting step, the material is solid with virtually no porosity. Reduction in porosity results in reduced layer thickness of the melted zones, i.e., volume shrinkage and an increase of the overall layer thickness up to 80 *μ*m [14]. This means that a powder with particle size of 45-106 *μ*m is suitable for applying a layer with a theoretical thickness of 50 *μ*m.

Hausner ratio (HR) and Carr's compressibility index were used to assess the flowability of the powder. Both of them were determined in accordance with EN ISO 3953 (ASTM D7481). Hausner ratio is defined as the ratio of tapped density of the powder (*V*_0_) to bulk density of the powder (*V*_f_) (1). The Carr's compressibility index (2) is presented. Formulas (1) and (2) are, respectively, calculated as follows:

(1) $$\begin{array}{c} \text{HR}=\frac{V_{0}}{V_{f}}, \end{array}$$

(2) $$\begin{array}{c} C=\frac{100\cdot \left(V_{0}-V_{f}\right)}{V_{0}}. \end{array}$$

Materials with Hausner ratio not higher than 1.25 and Carr's index not higher than 10 are suitable to be used in powder bed-based additive technologies [15]. Five tests were carried out for Ti-55511 alloy and the results were averaged. Hausner ratio (HR) for Ti-55511 alloy is 1.06, while Carr's index is 5.67, which means that the alloy falls in the first flowability class in respect of both indicators (excellent) [16, 17].

Bulk density was determined in accordance with EN ISO 3923-1:2010. This value is defined as the mass of the powder divided by the volume it occupies. It is calculated using the following formula:

(3) $$\begin{array}{c} \rho_{\text{nas}}=\frac{m_{\text{ps}}}{v_{\text{pl}}}\left[\frac{\text{g}}{{\text{cm}}^{3}}\right], \end{array}$$

where *m*_ps_ is the mass of an untapped powder sample in a cylinder [g], and *V*_pl_ is the volume of an untapped powder sample in a cylinder [cm^3^].

The test was carried out by passing 50 g of the tested material into a standardized and graduated cylinder. The measurement was repeated five times and the results were averaged. The determined bulk density of Ti-55511 alloy was (2.66 ± 0.5) g/cm^3^.

The starting points for calculating the energy required to melt the powder were the standard processing parameters for two-phase *α*+*β* titanium alloys. The required energy calculated using formula (4) was 40 J·mm^−3^.

(4) $$\begin{array}{c} E=\frac{I\cdot U}{V\cdot l_{\text{o}}\cdot l_{\text{t}}}\left[\frac{\text{J}}{{\text{mm}}^{3}}\right], \end{array}$$

where *U* is the accelerating voltage [V], *I* is the cathode current [mA], *V* is the scanning speed [mm/s], *l*_o_ is the spacing [mm], and *l*_t_ is the layer thickness [mm].

The initial measurement results for energy required to melt the *α*+*β* titanium alloy powder showed that it is lower than the theoretical energy, i.e., 25-30 J/mm^3^. Lower values result from Arcam EBM using automatic power control algorithms. The algorithm analyzes each layer and calculates cathode current and beam speed to provide enough energy to melt the powder layer. Thus, thermal stresses and vaporization of light alloying elements from the molten material are reduced.

Because the algorithms are not accessible to an average user, the actual values would not be scientifically relevant for the work described in this paper, and therefore, in our study, this functionality was turned off. This allowed for full control of the process parameters.

The following parameters and their impact were analyzed in the study:

- Energy input is 10 J/mm^3^-50 J/mm^3^. Formula (4) shows that the same energy input may be obtained for varying sets of parameters. Cathode current and scanning speed were used as variable parameters in the formula
- Cathode current is 4.5 mA-19.5 mA
- Scanning speed is 1080 mm/s-23400 mm/s. The speed was adjusted so that with a given cathode current, the set energy input value is obtained as per formula (4)
- The constant parameters used in the study were as follows:
  - Accelerating voltage is 60 kV
  - Distance between scan lines is 0.1 mm
  - Layer thickness is 0.05 mm

A total of 20 parameter sets were prepared (Table 2). Each set was repeated four times.

Specimens were used to analyze the impact of process parameters on the quality of the top surface, porosity, microstructure, and hardness.

Porosity was measured in four planes, i.e., three planes parallel and one plane perpendicular to the build direction. The proportion of pores within the area of a minimum of 12 mm^2^ was measured at 400x magnification. The average value derived from measurements was used to assess the effect of parameters on porosity.

The microstructure was examined on a plane parallel to the build direction. The fabricated specimens were cut, mechanically ground, polished, and then washed with ethanol. They were then etched with Kroll's reagent (2% HF, 5% HNO_3_, and 93% H_2_O). The microstructures of the specimens were observed using scanning electron microscope. Hardness was measured on a surface parallel to the build direction by the Vickers test using a 1 N load.

## 3. Results and Discussion

### 3.1. Top Surface Quality

**Figure 3:**
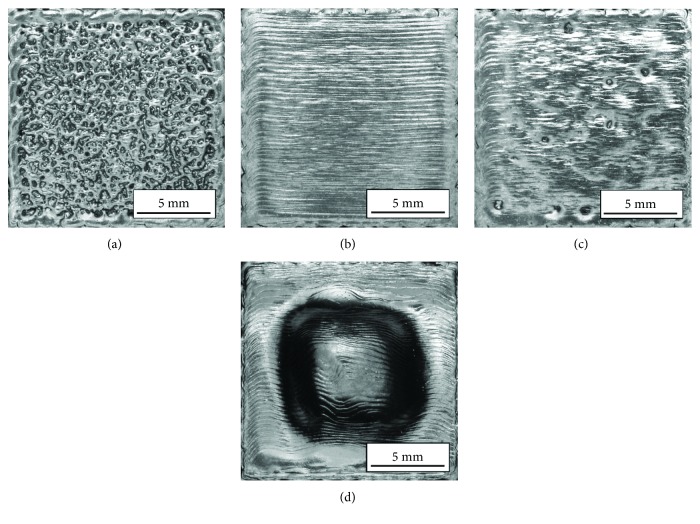
Different types of top surfaces of manufactured specimens: (a) orange peel, *I* = 4.5 mA and *V* = 5400 mm/s; (b) surface with single pores, *I* = 4.5 mA and *V* = 2700 mm/s; (c) the desired type of surface, *I* = 4.5 mA and *V* = 1800 mm/s; (d) surface with swelling, *I* = 14.5 mA and *V* = 3480 mm/s.

Observation of the specimen's top surface showed that energy input had the greatest impact on its quality. Depending on the energy input and the parameter set used, four different types of the top surfaces were obtained (Figure 3).

**Figure 4:**
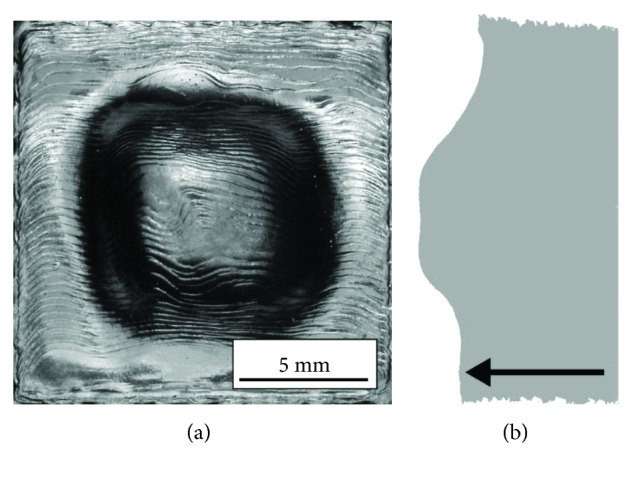
Specimen's surface with swelling: (a) top view and (b) side view. Arrow indicates build direction.

The first type of surface resembles an orange peel (Figure 3(a)). It is formed when the energy input is too low and was observed for parameter set nos. 1-4 (Table 2). The reason why this type of surface was formed was high scanning speed which resulted in the melted track losing its continuity [18]. The second type of surface has single small pores visible mainly in the central part of the specimen (Figure 3(b)). This type of surface was observed in three out of four sets of parameters, for energy of 20 J/mm^3^ (parameter set nos. 5, 6, and 7). The third type of surface without pores or orange peel (Figure 3(c)) is the desired one in terms of quality (parameter set nos. 9-14 and nos. 17 and 18). The last type of surface had pronounced swelling (Figure 3(d)) in the center of the specimen (Figure 4).

**Figure 5:**
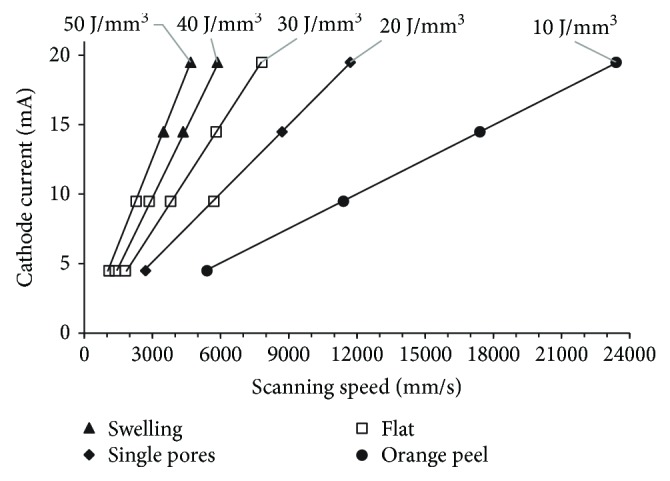
Influence of scanning speed and cathode current on top surface quality of EBM-fabricated specimens.

It was observed in specimens fabricated using two out of four sets of parameters for energy input of 40 J/mm^3^ (parameter set no. 15 and no. 16) and for energy input of 50 J/mm^3^ (parameter set no. 19 and no. 20).  Figure 5 shows the relationship between the type of top surface of the specimens and the process parameters.

The reason why swelling occurs in EBM-fabricated parts is not fully understood. Similar phenomena were observed in welds [19–22]. Swelling in welding is related to surface tension effects on a weld pool. In the case of EBM, swelling may be the result of the movement of liquid material [23] in the specimen due to overheating (associated with the zig-zag scanning strategy) and small dimensions of the specimen [24]. The high energy which is supplied to the specimen makes the moving electron beam not only melt the powder but additionally heat the material that had already been melted. Each subsequent shift of the beam results in the not fully solidified material additionally moving from the edge of the specimen to its center. The outer part of the specimen surrounded by the powder gives off more heat compared to the central part, so that less material is accumulated on the edges than in the central part.

### 3.2. Porosity

**Figure 6:**
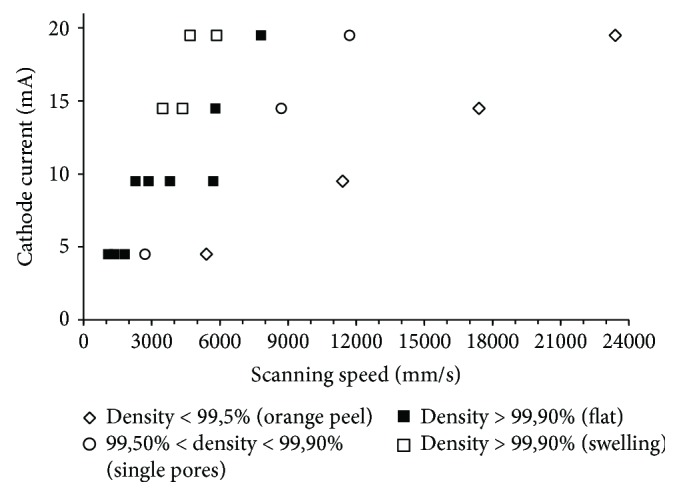
The specimens' relative density depending on the scanning speed and cathode current.

The results of porosity measurements showed that the volume fraction of pores in the fabricated specimens is between 0.02% (50 J/mm^3^, no. 20) and 11.18% (10 J/mm^3^, no. 4). The corresponding relative densities are 99.98% and 88.81%, respectively.  Figure 6 shows the correlation between the specimens' relative density and scanning speed and cathode current.

Comparison of the results shown in Figures 5 and 6 suggests that for some input energy ranges (40 J/mm^3^and 50 J/mm^3^) and scanning speeds, a high density in excess of 99.90% and a flat top surface can be obtained. Specimens with an orange peel surface had a low relative density (between 88.81 and 94.1%). Relative density of specimens with single pores on the top surface is ranged from 99.5% to 99.90%.

**Figure 7:**
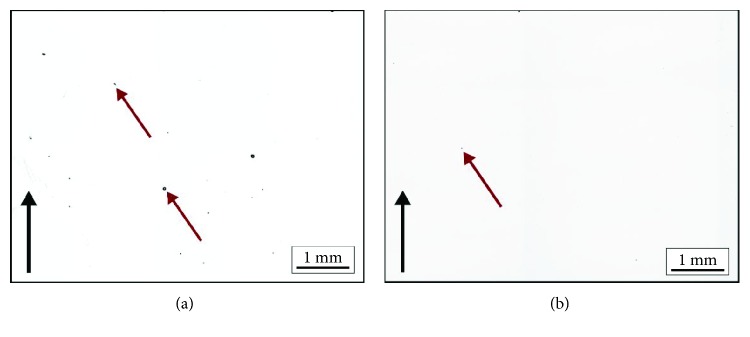
Size and distribution of pores for energy input of (a) 20 J/mm^3^, speed 11,700 mm/s and (b) 40 J/mm^3^, speed 4350 mm/s; red arrows indicate gas pores; black arrows indicate build direction.

For energy of above 20 J/mm^3^ and scanning speed below 5700 mm/s, the density of the specimens was over 99.5% (Figure 7(a)). Supplying energy of above 40 J/mm^3^ and reducing speed to less than 4680 mm/s produced densities in excess of 99.99% (Figure 7(b)).

**Figure 8:**
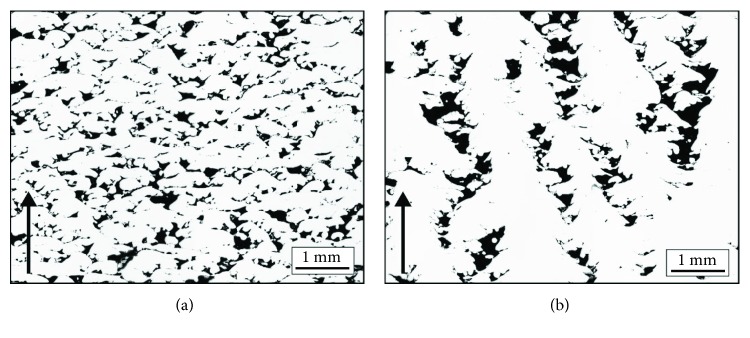
Porosity of the specimens fabricated with energy input of 10 J/mm^3^: (a) scanning speed of 5400 mm/s and (b) scanning speed of 23,400 mm/s.

In the specimens with a flat top surface, only gas pores were observed resulting from the release of gas trapped between the powder particles and probably of the powder itself. For the energy of 10 J/mm^3^, the density was between 88.81 and 94.1%. High porosity in excess of 5.0% resulted from the insufficient energy supplied to the material. Depending on the scanning speed, multilayer binding faults are obtained (Figure 8(a)), as well as channel-like porosities that propagate through layers (Figure 8(b)).

At low scanning speed (5400 mm/s), the melted track loses its continuity and melt pool dynamics is driven mostly by capillary forces and wetting causing surface tension of the melt [18, 25]. The low scanning speed also results in longer melting time of a layer and thus in reduced temperature and energy supplied to the material. In the case of higher scanning speeds (23,400 mm/s), the scanning dynamics reduced the temperature to a lesser extent, thus changing the nature of the pores from multilayer binding faults to channel-like ones.

### 3.3. Microstructure

**Figure 9:**
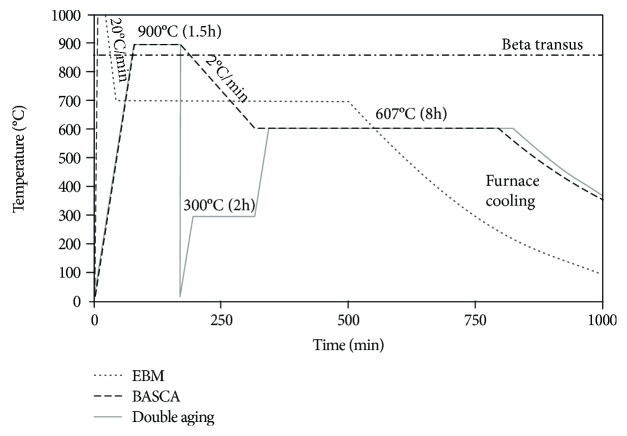
Standard heat treatment temperature profiles of BASCA and DA for *β* and near-*β* titanium alloys [30] in comparison with EBM temperature profile.

Ti-55511 alloy has two main types of microstructure, namely, lamellar or equiaxed. Equiaxed microstructure is formed after deformation below beta-transus temperature *T*_B_ in the range of two-phase field. This kind of microstructure consists of the globular *α*-phase dispersed in *β*-phase matrix [26, 27]. Typical heat treatments (HT) for *β*-phase and near-*β*-phase alloys are solution treatment and ageing (STA) [28], double aging (DA) [29], and beta annealing followed by slow cooling and aging (BASCA) [31]. In the case of STA, heat treatment process parameters, temperature, and time depend on the initial state and the microstructure of the processed material. Two types of HT are usually used for forged parts: the first one is solution treatment at 750-860°C/1 h, air or water quenching and DA (first ageing at 300°C/2 h, second at 495-667°C/8 h) and the other one is BASCA. Both of them are shown in Figure 9.

**Figure 10:**
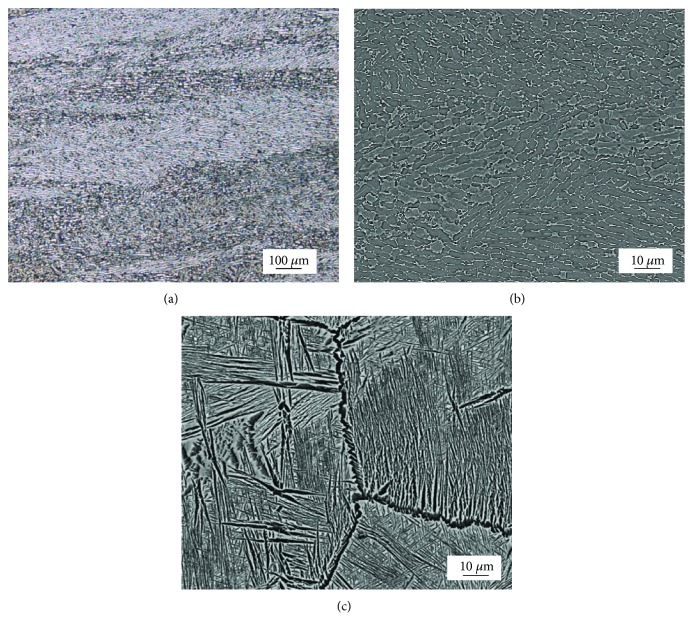
Microstructures of Ti-55511 alloy under two conditions: (a, b) cast by VAR method and hot rolled and (c) after BASCA heat treatment [30].

Figure 10 shows the reference Ti-55511 alloy. The specimen was processed by the vacuum arc remelting (VAR) casting methods and hot rolled.  Figure 10(a) shows the directionality of the structure related to rolling direction. The higher magnification image (Figure 10(b)) revealed that microstructure consists of equiaxed grains.  Figure 10(c) shows the microstructure after BASCA heat treatment. The specimen consists of lamellar structure formed when heat treatment or deformation occurs above the beta-transus temperature *T*_B_ with additional slow cooling. The last depicted microstructure consists of colonies of hexagonal close packed (hcp) *α*-phase lamellae within large (bcc) *β*-phase grains of several hundred microns in diameter [32].

**Figure 11:**
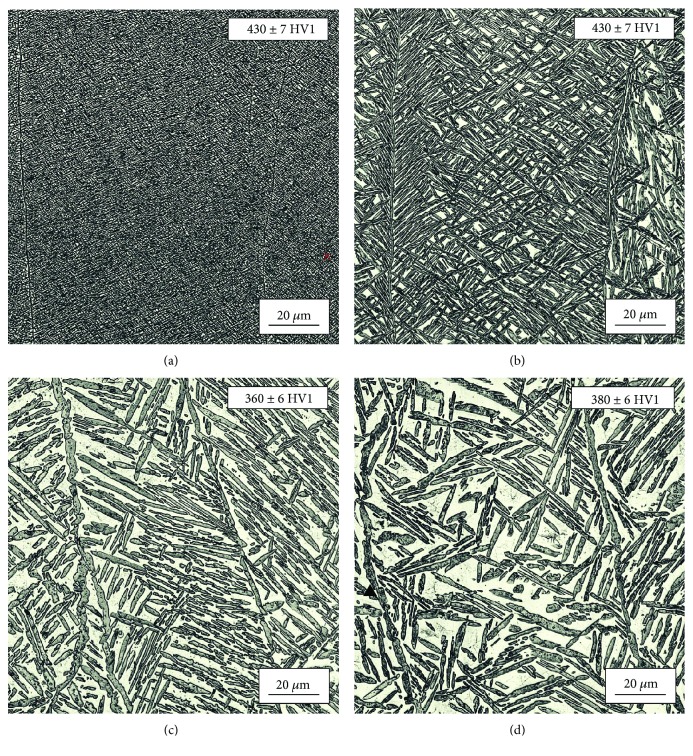
Microstructure of the specimens fabricated with the same energy input (30 J/mm^3^) and at different scanning speeds, i.e., (a) 1800 mm/s, (b) 3800 mm/s, (c) 5800 mm/s, and (d) 7800 mm/s. Arrows indicate build direction.

**Figure 12:**
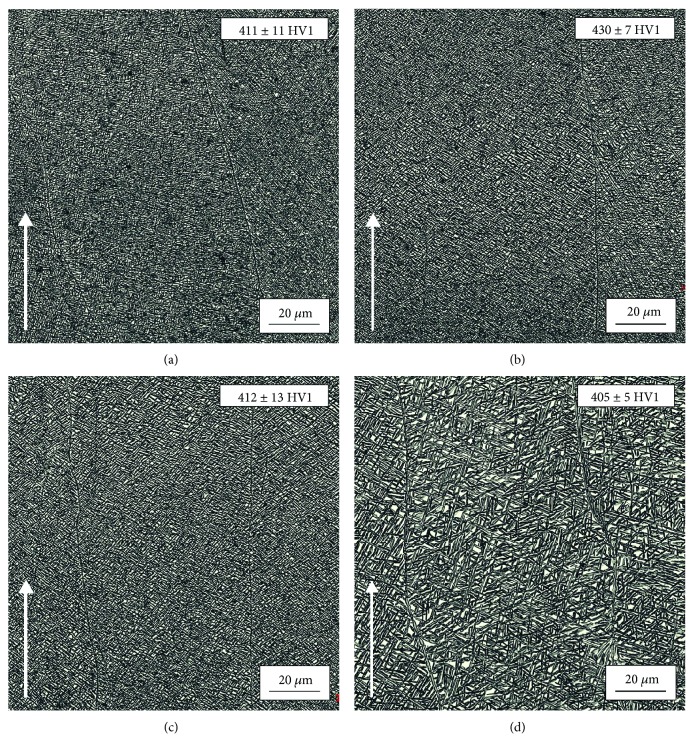
Microstructure of the specimens fabricated with different energy inputs and with the same cathode current (4.5 mA): (a) 20 J/mm^3^, (b) 30 J/mm^3^, (c) 40 J/mm^3^, and (d) 50 J/mm^3^; arrow indicates build direction.

The images of microstructure of EBM-fabricated Ti-55511 alloy (Figures 11 and 12) show the microstructure similar to that shown in Figure 10(c). Comparison of the typical heat treatment for *β* and near-*β* titanium alloys and EBM process suggests that during EBM process the specimens solidify and are cooled down to about 600°C-650°C and then are held at this temperature for about 2-10 hours, depending on the time of the fabrication process [33]. The final stage of EBM process is cooling down of the entire platform (with the fabricated specimens and sintered powder). These conditions are suitable for the nucleation and growth of the *α*-phase in the *β*-phase. The cooling rate and platform temperature differ for individual EBM processes and depend primarily on the process parameters used and the volume of the specimens built. Nucleation of the *α* from the *β* starving regions was seen to occur at 500°C after only 0.1 h [34].

The microstructures of specimens fabricated at different scanning speeds are shown in Figure 11. The specimens were manufactured with varying energy input, scanning speed, and cathode current. Figure 11 shows a very strong impact of the scanning speed on the microstructure obtained. Higher scanning speed results in a reduced amount of the *α*-phase. For scanning speed of 1800 mm/s, the amount of thin lamellar *α*-phase was approximately 78% (measured by counting the pixels per *α*-phase lamellas). As the scanning speed increased, the *α*-phase amount was reduced to 61% (at 7800 mm/s). The dimensions of the lamellas and the amount of the *α*-phase strongly influenced hardness measurement results. The highest hardness (430 ± 7 HV1) was measured for the specimen fabricated at scanning speed of 1800 mm/s, characterized by the smallest lamellas and about 78% amount of the *α*-phase. Higher scanning speed reduced hardness. The specimen fabricated at scanning speed of 5800 mm/s with relatively large lamellas and about 65% amount of the *α*-phase had the lowest hardness of 360 ± 6 HV1. Interestingly, a further increase of the scanning speed did not reduce hardness any further (380 ± 10 HV for the specimen fabricated at scanning speed of 7800 mm/s). This is most likely due to application of a very high cathode current of 20 mA for this specimen.

Differences in microstructure were also observed for varying energy inputs (Figure 12). However, they are not as pronounced as for the varying scanning speed parameter. All specimens had a very fine *α* + *β*_M_ lamellar structure with slightly visible columnar grain boundaries of the primary *β*-phase. This phase forms by epitaxial growth corresponding to the specimen's build direction (indicated by an arrow). Images of the specimens fabricated with a higher energy input show that as the energy increases, dispersion of lamellas decreases. The thickest lamellas were observed in the specimens fabricated with the highest energy input, i.e., 50 J/mm^3^ (Figure 12(d)). Differences in hardness are not as significant as for varying scanning speed (Figure 11). The lowest hardness (405 ± 5 HV1) was obtained for the specimen fabricated with energy input of 50 J/mm^3^. The highest hardness (430 ± 7 HV1) was measured for the specimen fabricated with energy input of 30 J/mm^3^.

**Figure 13:**
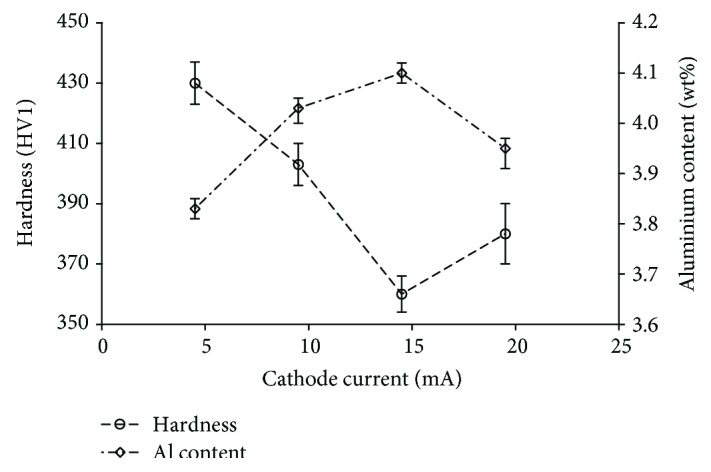
Hardness and aluminum content versus cathode current. Energy input of 30 J/mm^3^. For aluminum content, the error bars represent the standard deviation of measurements for 4 points, 6 measurements per point. For hardness, the error bars represent the standard deviation of measurements for 4 points, 10 measurements per point.

Figure 13 shows the averaged hardness test results for the specimens fabricated at different scanning speeds and different cathode current values. The highest hardness (430 ± 7 HV1) was obtained for the specimen fabricated at scanning speed of 1800 mm/s and with cathode current of 4.5 mA. The lowest hardness (360 ± 6 HV1) was measured for the specimen fabricated at 5800 mm/s and with cathode current of 14.5 mA.

**Figure 14:**
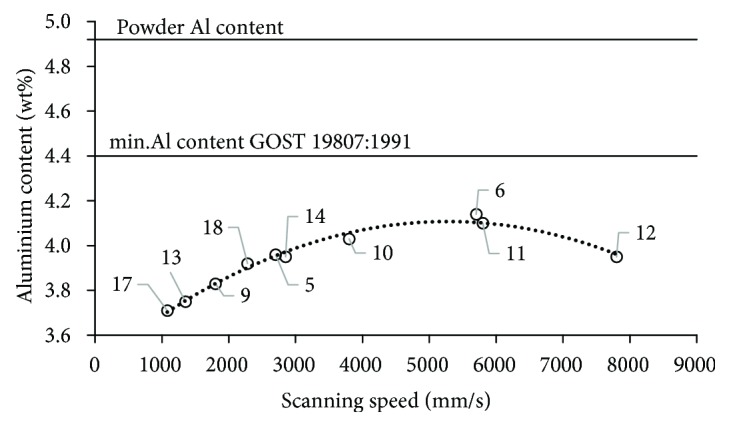
Aluminum content in EBM-fabricated specimens versus scanning speed. Numbers represent parameter sets.

One of the characteristic features of generative technologies is that some elements partially vaporize during powder processing. The extent to which individual alloying elements vaporize in the powder material may significantly modify the chemical composition of the fabricated specimen. Therefore, a control analysis of the chemical composition of the specimens produced was carried out by energy-dispersive spectrometry (EDS). The analysis showed that significant vaporization of aluminum was observed in the course of EBM process. Figure 13 shows hardness test results and the corresponding results of the aluminum content analysis. A similar aluminum vaporization phenomenon was observed by Lach et al. [35] who reported a very pronounced aluminum loss in Ti-47Al-2Cr-2Nb alloy processed by selective electron beam melting (SEBM). Lach et al. [35] found that the greatest Al loss (by 15%) occurred for high energy input. The analysis results presented in Figure 14 show that aluminum vaporized most in the specimens fabricated at the lowest scanning speed (3.7% Al for the specimen fabricated at 1080 mm/s).

**Figure 15:**
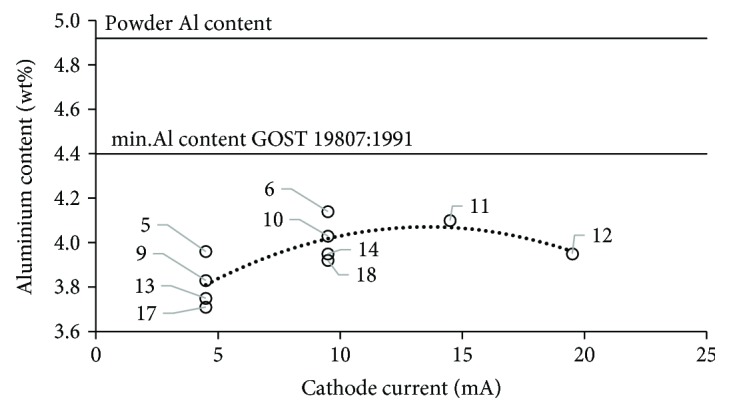
Aluminum content in EBM-fabricated specimens versus cathode current. Numbers represent parameter sets.

Aluminum content increases with increased scanning speed to reach the maximum (4.1 wt% Al) for scanning speed of 5700 mm/s. When the speed was further increased (7800 mm/s), Al content decreased to 3.9 wt%. The results published by Brice et al. [36] showed that the aluminum content dropped significantly also in the samples made from Ti-6Al-4V alloy processed by EBM. This is due to the fact that aluminum has higher vapor pressure than titanium and vanadium and thus vaporizes preferentially. The results shown in Figure 14 reveal that in all of the specimens tested, there also was a pronounced aluminum loss in comparison to the powder used whose aluminum content was 4.9 wt% (Table 1).  Figure 15 shows the changes in aluminum content versus cathode current.

Aluminum content increases with the increase of cathode current to around 15 mA (aluminum loss decreases), and once this value is exceeded, it begins to decrease. This parameter has an impact on aluminum loss, but not as strong as scanning speed. As can be seen in Figure 15, aluminum vaporization may vary considerably for the same cathode current. Aluminum is the element stabilizing the *α*-phase in titanium alloys, which means that it increases the *α*↔*β* transformation temperature. Aluminum loss in Ti-55511 alloy may therefore induce reduction of the precipitation temperature of the *α*-phase, depending on the size of the loss. This suggests that aluminum loss combined with the strong influence of the cooling rate determined by the process parameters results in a high degree of fineness of the *α*-phase lamellas and thus in increased hardness.

## 4. Conclusions

The test results indicate that EBM technique can be successfully applied to process one of the Ti-5Al-5Mo-5V-1Cr-1Fe (Ti-55511) near-*β* titanium alloys. The tests have shown that it is possible to determine the process parameter window to fabricate specimens with relative density in excess of 99%, with flat free surfaces without pores, orange peel, or deformation. The highest quality specimens with relative density in excess of 99.9% and flat surfaces are obtained with energy density of 30 J/mm^3^, regardless of the corresponding cathode current and scanning speed settings. The microstructure of Ti-55511 alloy fabricated by EBM consists of the lamellar *α*-phase distributed in the matrix of the primary *β*-phase. The volume fraction of the *α*-phase and its dispersion depend on EBM parameters used, i.e., scanning speed and energy density. The higher the fragmentation and the relative amount of the *α*-phase, the higher the hardness of the alloy. The maximum hardness (430 ± 7 HV1) was measured for Ti-55511 alloy with an 80% amount of the *α*-phase processed using energy input of 30 J/mm^3^ and scanning speed of 1800 mm/s. Variations in the chemistry of Ti-55511 alloy due to the vaporization of aluminum are the negative effect of EBM processing. The amount of aluminum loss depends mainly on the scanning speed: the lower the speed, the higher the Al loss. This important phenomenon must be taken into account in the process of designing and fabricating parts from titanium alloys containing aluminum.

## Figures and Tables

**Table 1:** Chemical composition of Ti-55511 alloy according to GOST 19807:1991 [13] and the certificate provided by the manufacturer.

	Ti	Al	Mo	V	Cr	Fe
	(wt%)					
Reference	Balance	4.4-5.7	4.0-5.5	4.0-5.5	0.5-1.5	0.5-1.5
Powder	Balance	4.92	5.09	4.97	1.02	1.02

**Table 2:** Sets of parameters used in the study.

No.	Energy input (J/mm^3^)	Cathode current (mA)	Scanning speed (mm/s)
1	10	4.5	5400
2		9.5	11,400
3		14.5	17,400
4		19.5	23,400

5	20	4.5	2700
6		9.5	5700
7		14.5	8700
8		19.5	11,700

9	30	4.5	1800
10		9.5	3800
11		14.5	5800
12		19.5	7800

13	40	4.5	1350
14		9.5	2850
15		14.5	4350
16		19.5	5850

17	50	4.5	1080
18		9.5	2280
19		14.5	3480
20		19.5	4680

## Data Availability

The data used to support the findings of this study are available from the corresponding author upon request.
